# Supplementary material for: Conformer-dependent vacuum ultraviolet photodynamics and chiral asymmetries in pure enantiomers of gas phase proline
Source: Commun Chem. 2021 May 20;4:72. doi: 10.1038/s42004-021-00508-z (PMC9814706; doi:10.1038/s42004-021-00508-z)
Supplement: Supplementary file 2 — Supplementary Information [file 42004_2021_508_MOESM2_ESM.pdf]

Conformer-dependent vacuum ultraviolet photodynamics and chiral asymmetries in pure enantiomers of gas phase proline

Rim Hadidi,[1] Dušan K. Božanić,[1, #] Hassan Ganjitabar,[2] Gustavo A. Garcia,[1] Ivan Powis,[2] Laurent Nahon[1] *

[1] *Synchrotron SOLEIL, l'Orme des Merisiers, Saint Aubin BP 48, 91192 Gif sur Yvette Cedex, France.*

[2] *School of Chemistry, The University of Nottingham, University Park, Nottingham NG7 2RD, UK*

[#]Present address: Department of Radiation Chemistry and Physics, "VINČA" Institute of Nuclear Sciences - National Institute of the Republic of Serbia, University of Belgrade, 11001 Belgrade, Serbia

*corresponding author: laurent.nahon@synchrotron-soleil.fr

Supplementary Table S1. **Summary of the conformer labelling, with the corresponding geometrical features) of the 4 main conformers of Pro considered in this work and in the literature**.

Conformer labelling (this work)		Czinki et al.[1]	Tian et al.[2] (a)	Mata et al.[3]	Fathi et al.[4] (a)	Lu et al.[5]	Pucker	H-bond	
II	A	I	Ia	IIa	IIa	Pro4	up	OH..N	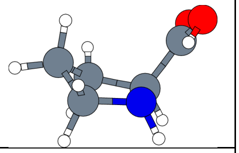
	B	II	Ib	IIb	IIb		down	OH..N	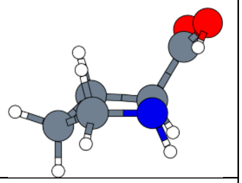
I	C	IV	IIb	Ib	Ia		down	NH..O	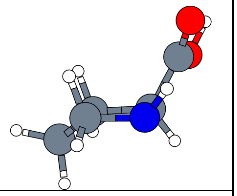
	D	III	IIa	Ia	Ib	Pro5	up	NH..O	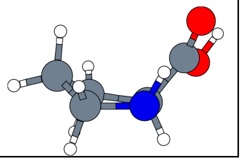

(a) NB These authors base their geometric analysis on the D-proline enantiomer

Supplementary Table S2. Heavy atom population analysis of the HOMO orbitals for Proline conformers A–D, based on the HF density in MP2/Aug-cc-pVDZ calculations. (H atoms and contributions <2% are excluded). The heavy atoms labelling is shown in the last column

Conf.		Atom							
		C_1	C_2	C_3	N_4	C_5	C_6	O_7	O_8
II	A		0.03*p*	0.07*p*	0.39*p*<br>0.03*s*	0.03*p*	0.03*p*	0.05*p*	0.23*p*
	B	0.03*p*	0.03*p*	0.06*p*	0.38*p*<br>0.04*s*	0.02*p*	0.02*p*	0.05*p*	0.26*p*
I	C			0.02*p*	0.61*p*<br>0.08*s*	0.05*p*	0.03*p*		
	D	0.06*p*			0.63*p*<br>0.08*s*	0.04*p*			

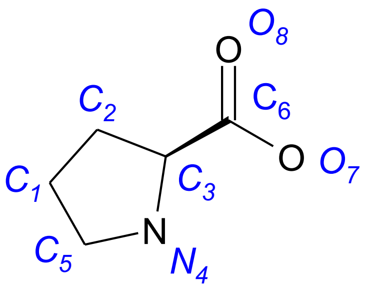

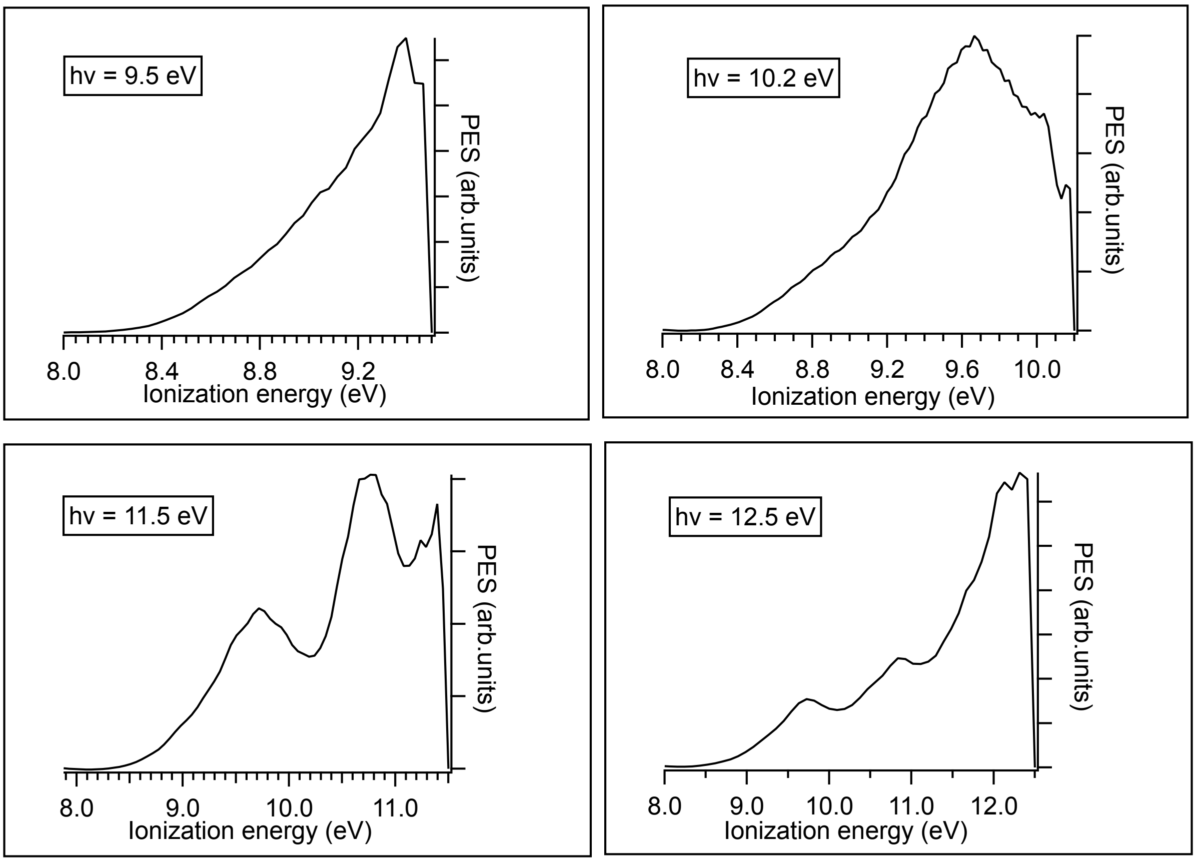

Supplementary Figure S1. PES of Pro (all masses) recorded at different photon energies ranging from 9.5 eV to 12.5 eV (TD_{415} condition).

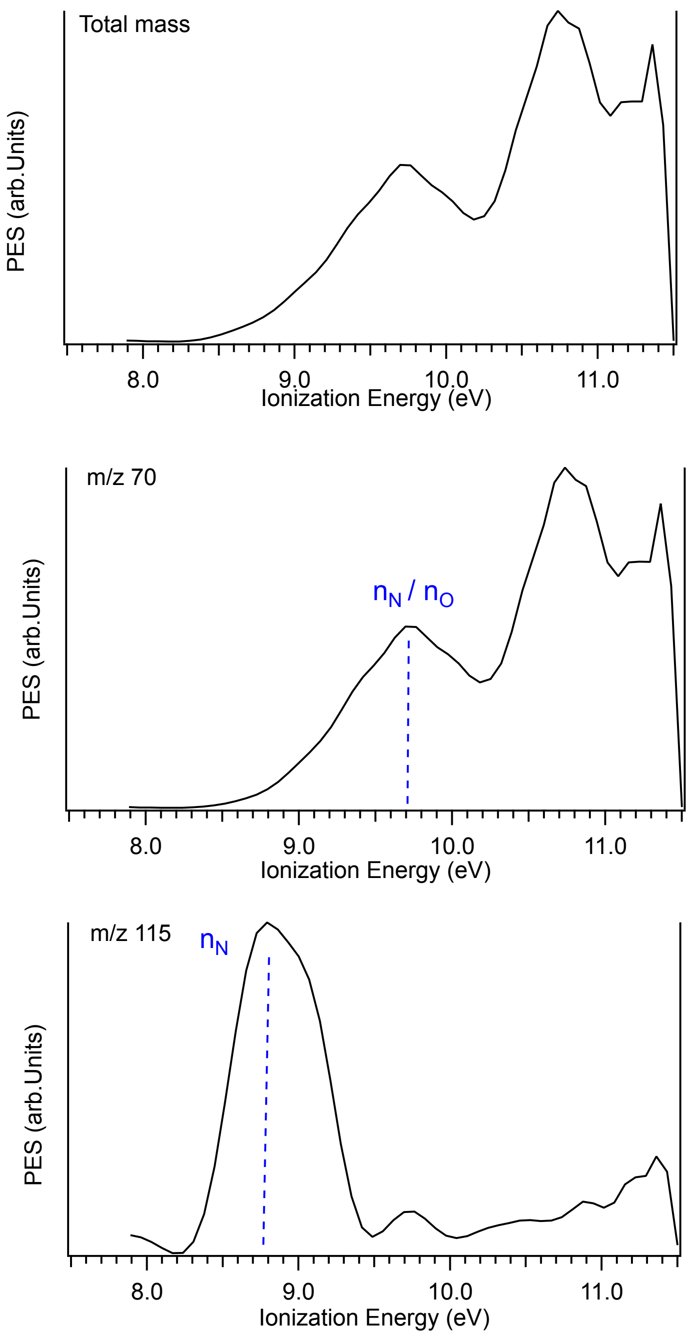

Supplementary Figure S2. **PES of Pro at hv= 11.5 eV filtered on the total mass, on the parent (*m/z* 115) and on the fragment (*m/z* 70).**

Supplementary Table S3. Calculated vertical ionization energies (in eV) for the 4 outermost orbitals of Proline

Band	Orbital	This work OVGF/cc-pVTZ//MP2/cc-pVTZ		OVGF[a]		B3LYP[b]		MP[c]	
		Conf I (C/D)	Conf II (A/B)	Conf I	Conf II	Conf I	Conf II	Conf I	Conf II
1	HOMO	9.05<br>9.02	9.58<br>9.72	8.75	9.36	8.83<br>8.71	9.41<br>9.52		8.81<br>8.941
2	HOMO-1	10.90<br>10.95	10.78<br>10.67	10.76	10.57	10.67<br>10.62	10.51<br>10.45		9.91<br>9.842
3	HOMO-2	12.16<br>11.96	11.29<br>11.36	11.7	10.88	11.86<br>11.95	11.15<br>11.17		10.66<br>10.737
4	HOMO-3	12.06<br>12.16	12.48<br>12.59	12	12.3	12.04<br>11.97	12.25<br>12.34		11.79<br>11.771

[a] Ref.6

[b] Ref.2

[c] Ref.4

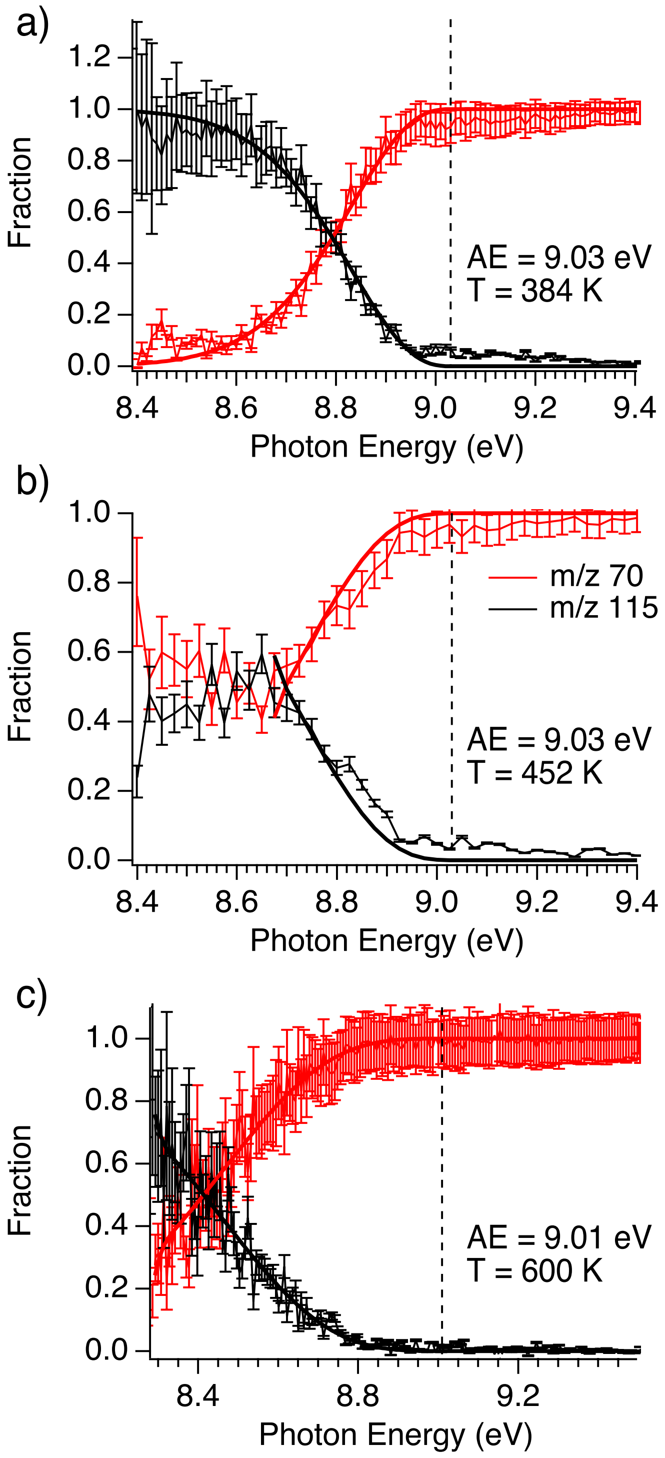

Supplementary Figure S3. Proline breakdown diagram recorded in the TD_{415}, (a), TD_{493} (b) and RH (c) conditions showing the dissociation of the parent ion (black curve) into the *m/z* 70 fragment (red curve). Errors bars correspond to statistical (standard deviation) error bars. The solid lines represent the fit to a statistical model. The dashed line shows the 0 K Appearance Energy of the *m/z* 70 fragment as derived from the fit. The quality of the data for the TD_{493}method prevented us from fitting the AE, which as then been frozen in the corresponding fitting.

Supplementary Table S4. Calculated MP2/aug-cc-pVDZ geometries of the four main conformers of proline neutral and the HOMO^{-1} cation. (see Table S2 for the heavy atoms labelling)

Conformer		H-bond length (Å)		Dihedral angle $N_4C_3C_6=O_8$ (°)		Dihedral angle $C_5N_4C_3C_6$ (°)	
		neutral	cation	neutral	cation	neutral	cation
II	A	1.83	2.70	-177.7	-7.1	116.5	102.0
	B	1.86	2.23	-172.2	8.8	107.5	140.9
I	C	2.43	2.32	-5.0	8.9	93.7	142.0
	D	2.22	2.68	-15.6	0.5	120.1	105.5

Supplementary Note 1. Production and characterization of gas phase proline.

Time of flight (TOF) mass spectra of Pro photoionized at different photon energies (hν), ranging from 8.7 eV to 17.5 eV were recorded (Figure S4), with the TD at two temperatures of 415 K and 493 K and with the oven at T= 468 K.

At 8.7 eV, the parent Pro at *m/z* 115 is predominant at all temperatures used, because mainly the conformers leading to a stable cation are photoionized. When the photon energy increases to 9.5 eV, the parent become less abundant due to the population of states leading to fragmentation, giving birth to a major fragment at m/z 70, produced by the cleavage of the C-Cα bond,[7] *i.e.* a loss of the carboxyl moiety, a typical fragmentation pattern already reported for other amino-acids[8-12] after valence-shell ionization in the gas phase. Above 10.2 eV, a peak at *m/z* 43 is observed as well as peaks around m/z 28-30 above 11.5 eV. These fragments have been observed in previous works,[7,13,14] according to which their formation could take place via different dissociation mechanisms involving a pyrrolydine ring opening. Except for a peak at *m/z* 18 visible at 13.5 eV for the TD_{493} case (Fig.S4(b)), attributed to H_2O present as a background compound in the chamber, all the species visible on the spectra recorded with the TD method are related to neutral nascent Pro.

This is not the case for the TOF spectra recorded with the RH method. Indeed, the better mass resolution compared to the TD, allows us to identify the presence of extra narrow peaks at *m/z* 67, 69, 71 and 72, assigned to thermal decomposition products (see Fig. 12), indicating a neutral decomposition in the oven prior to the adiabatic expansion, while the peak at *m/z* 70 appears broad because of the kinetic energy release (KER) imparted by dissociative ionization from nascent intact neutral Pro. At higher photon energies, above 12.5 eV, many peaks are appearing on the RH-TOFs in the m/z 20-50 range arising from dissociative ionization of deeper valence orbital of Pro as well as possibly from spurious compounds. Their analysis is beyond the scope of this work, and we consider here, and select via our PEPICO scheme, the parent (*m/z* 115) and the main fragment (*m/z* 70) originating from the ionization of the two outmost orbitals HOMO and HOMO-1.

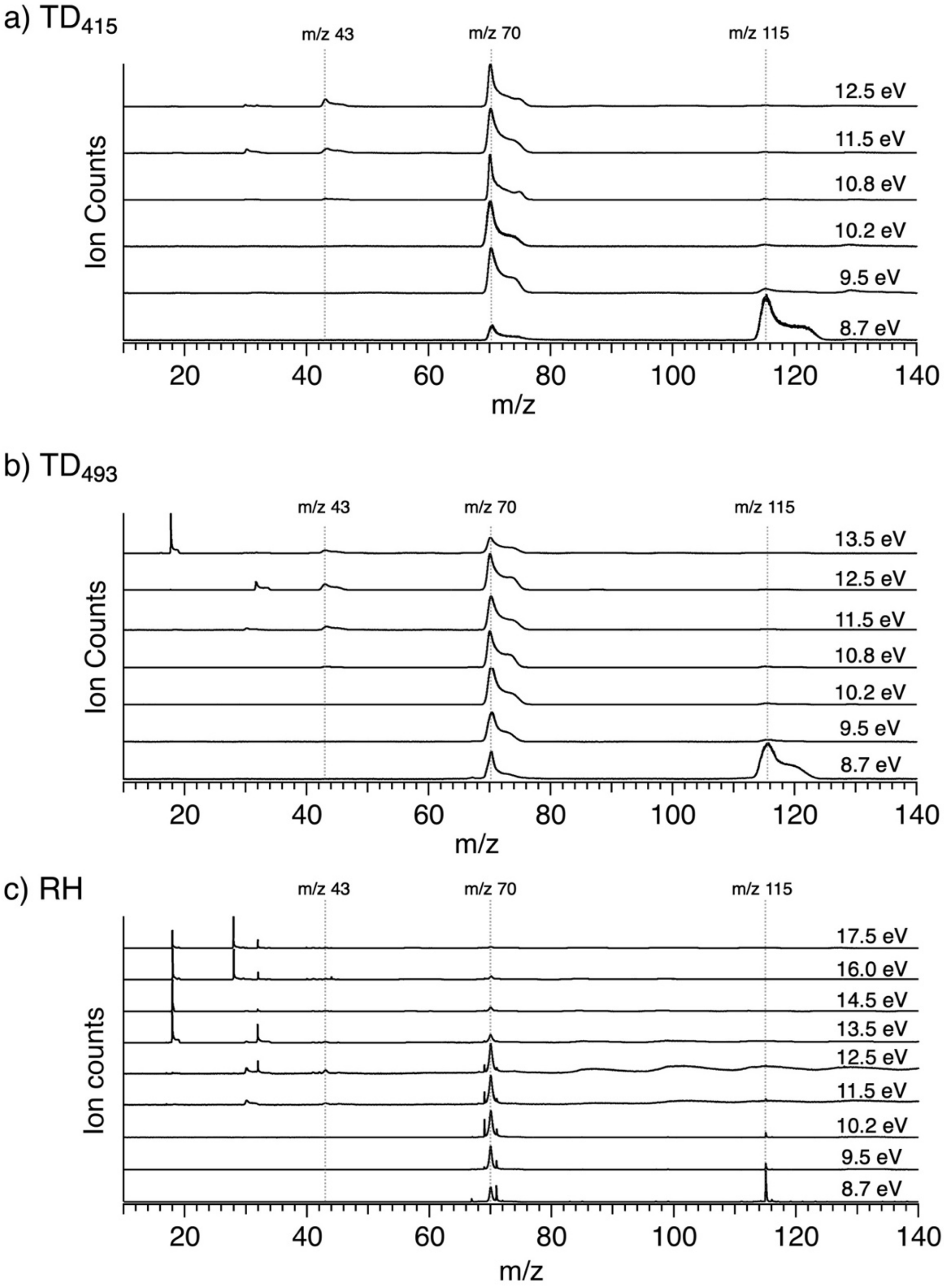

Supplementary Figure S4. Time-of-flight mass spectra (TOF) of proline recorded at different photon energies. **a** TD_{415} condition. **b** TD_{493} condition. **c** RH condition. The RH TOFs up to 11.5 eV have been ROI-filtered to remove the thermal background contribution. condition

Supplementary References

1 Czinki, E. & Császár, A. G. Conformers of Gaseous Proline. *Chemistry – A European Journal* **9**, 1008-1019, (2003).

2 Tian, S. X. & Yang, J. Effects of intramolecular hydrogen bonding on the ionization energies of proline. *Angew. Chem. Int. Ed. Engl.* **45**, 2069-2072, (2006).

3 Mata, S. *et al.* Observation of two new conformers of neutral proline. *Phys Chem Chem Phys* **11**, 4141-4144, (2009).

4 Fathi, F. & Farrokhpour, H. Valence ionization of l-proline amino acid: Experimental and theoretical study. *Chem. Phys. Lett.* **565**, 102-107, (2013).

5 Lu, J., Meng, D., Li, F., Guo, M. & Li, Y. Theoretical Study of the Structure and Ionization Potentials of Proline. *Russian Journal of Physical Chemistry A* **94**, 1427-1432, (2020).

6 Dehareng, D. & Dive, G. Vertical ionization energies of alpha-L-amino acids as a function of their conformation: an ab initio study. *International Journal of Molecular Sciences* **5**, 301-332, (2004).

7 Coutinho, L. *et al.* Photoabsorption and photoionization studies of the amino acid proline in the VUV region. *Brazilian Journal of Physics* **35**, 940-944, (2005).

8 Tia, M. *et al.* Chiral asymmetry in the photoionization of gas-phase amino-acid alanine at Lyman-a radiation wavelength. *J. Phys. Chem. Lett.* **4**, 2698-2704, (2013).

9 Tia, M. *et al.* VUV photodynamics and chiral asymmetry in the photoionization of gas phase alanine enantiomers. *J. Phys. Chem. A* **118**, 2765-2779, (2014).

10 Pan, Y. *et al.* Intramolecular hydrogen transfer in the ionization process of alpha-alanine. *Phys. Chem. Chem. Phys.* **11**, 1189-1195, (2009).

11 Simon, S., Gil, A., Sodupe, M. & Bertrán, J. Structure and fragmentation of glycine, alanine, serine and cysteine radical cations. A theoretical study. *Journal of Molecular Structure: THEOCHEM* **727**, 191-197, (2005).

12 Laksman, J. *et al.* Dissociation Pathways in the Cysteine Dication after Site-Selective Core Ionization. *The Journal of Physical Chemistry B* **118**, 11688-11695, (2014).

13 Lago, A. F., Coutinho, L. H., Marinho, R. R. T., de Brito, A. N. & de Souza, G. G. B. Ionic dissociation of glycine, alanine, valine and proline as induced by VUV (21.21 eV) photons. *Chem. Phys.* **307**, 9-14, (2004).

14 Tamuliene, J. *et al.* On the influence of low-energy ionizing radiation on the amino acid molecule: proline. *The European Physical Journal D* **70**, 143, (2016).
